# Biochemical properties and yields of diverse bacterial laccase-like multicopper oxidases expressed in *Escherichia coli*

**DOI:** 10.1038/srep10465

**Published:** 2015-06-12

**Authors:** Julian Ihssen, Renate Reiss, Ronny Luchsinger, Linda Thöny-Meyer, Michael Richter

**Affiliations:** 1 Empa, Swiss Federal Laboratories for Materials Science and Technology, Laboratory for Biointerfaces, Lerchenfeldstr.5, 9014 St. Gallen, Switzerland

## Abstract

Laccases are multi-copper oxidases that oxidize a broad range of substrates at the expense of molecular oxygen, without any need for co-factor regeneration. These enzymes bear high potential for the sustainable synthesis of fine chemicals and the modification of (bio)polymers. Here we describe cloning and expression of five novel bacterial laccase-like multi copper oxidases (LMCOs) of diverse origin which were identified by homology searches in online databases. Activity yields under different expression conditions and temperature stabilities were compared to three previously described enzymes from *Bacillus subtilis, Bacillus pumilus* and *Bacillus clausii*. In almost all cases, a switch to oxygen-limited growth conditions after induction increased volumetric activity considerably. For proteins with predicted signal peptides for secretion, recombinant expression with and without signal sequence was investigated. *Bacillus* CotA-type LMCOs outperformed enzymes from *Streptomyces* and Gram-negative bacteria with respect to activity yields in *Escherichia coli* and application relevant biochemical properties. The novel *Bacillus coagulans* LMCO combined high activity yields in *E. coli* with unprecedented activity at strong alkaline pH and high storage stability, making it a promising candidate for further development.

Laccases (EC 1.10.3.2) are the largest subgroup of the protein superfamily of multi-copper oxidases (MCOs) and catalyse the one electron oxidation of four substrate molecules at the expense of one molecule dioxygen^1^. Substrate oxidation takes places at the mono-nuclear copper center (T1); electrons are then transferred to the tri-nuclear copper center (T2/T3), where the reduction of dioxygen takes place, yielding water as the sole by-product^2^. Laccases have a broad substrate spectrum ranging from substituted phenols and polyphenols over heterocyclic compounds to inorganic compounds such as iodide^3^,^4^,^5^. This property and the ease of use of these enzymes have generated a high industrial interest in laccases, particularly within in the wood, biofuel, paper, textile, fine chemicals and food sectors. Laccases or laccase-like enzymes have been isolated from plants, fungi and bacteria, and corresponding signature sequences have been found in numerous genome sequences of eukaryotic and prokaryotic organisms through bioinformatic analysis^6^,^7^,^8^. To date, the discrimination of laccases from other MCOs is still ambiguous. The strategy to find novel laccases mostly focuses on identifying the strictly conserved histidine residues of the copper binding motifs by homology searches, usually followed by the experimental proof of some kind of phenol oxidase activity. As laccases were first discovered in the sap of the lacquer tree *Rhus vernicifera*, it was suggested that only MCOs isolated in the presence of the natural substrate urushiol, an unsaturated alkyl catechol, should be classified as laccases. The term “laccase-like MCOs” (abbr. LMCO) was proposed for all other enzymes that have previously been classified as laccases^7^. We agree with this classification, especially in terms of bacterial homologues and therefore use the term LMCO in this report.

Due to difficulties in expressing fungal LMCOs in bacteria, production strategies rely on natural producer organisms or heterologous expression in eukaryotic hosts such as *Pichia pastoris*, *Saccharomyces cerevisiae, Aspergillus* spp. and *Trichoderma* spp.^9^,^10^,^11^,^12^,^13^. Yeast expression systems suffer from low yields, and the production time with filamentous fungi is usually considerably longer than with yeasts^14^. Bacterial LMCOs can be expressed in *Escherichia coli,* probably still the most favored expression system for recombinant proteins, in particular for industrial biocatalysts. Various approaches for increasing the yield of active recombinant enzymes in *E. coli* have been described^15^. Expression levels can be strongly enhanced by using high copy number plasmids with strong promoters, e.g. plasmids of the pET series. Alternatively, protein expression can be improved by eliminating rare codons or by using special strains with additional copies of rare tRNAs^16^,^17^. The sequence of synthetic genes can be adapted to the codon bias of the host by specific algorithms^18^. However, the use of strong promoters and removal of rare codons does not necessarily lead to the highest levels of active protein, because a large part of the overexpressed polypeptide may end up incorrectly folded in insoluble inclusion bodies^19^,^20^,^21^. The presence or absence of secretory signal peptides can also have profound effects on solubility and activity of heterologous enzymes in *E. coli*^22^. Furthermore, in the case of oxidoreductases the correct insertion of (metal) co-factors is a crucial step for obtaining active enzyme.

Various bacterial LMCOs have been expressed in *E. coli*^23^,^24^,^25^. As LMCOs require 4 copper ions for catalysis, a sufficient supply of copper ions in the cytoplasm is crucial for obtaining active enzyme. However, *E. coli* cells prevent intracellular accumulation of toxic copper with inducible efflux systems^26^. Cells grown under anoxic conditions do not induce these systems to the same extent. Thus, for the production of recombinant CotA LMCO from *Bacillus subtilis,* cultivation conditions were successfully adapted to increase intracellular copper levels by simply introducing a static cultivation step after induction, resulting in fully copper loaded enzyme^27^. The same procedure was applied for the efficient expression of *Bacillus pumilus* CotA in *E. coli*^28^.

In the present study activity yields and biochemical properties were determined for phylogenetically diverse LMCOs of bacterial origin. The effect of signal peptides and co-expression of rare tRNA was analysed. Kinetic parameters, substrate range, pH range and storage stability were determined for a promising, novel CotA-type LMCO derived from *Bacillus coagulans*.

## Results

### Identification and description of analysed LMCOs

An increased use of LCMOs in industrial processes requires access to a high number of efficiently produced (recombinant) enzymes of this class^29^. Ideally, to match the criteria of platform biocatalysts for technically relevant processes the set of available LMCOs should cover a broad substrate range and preference, show heat, solvent and storage stability and have activity over a broad pH range. In an attempt to find novel bacterial LMCOs which can be produced in *E. coli*, we performed an extensive database search using protein BLAST. The protein sequences of *B. subtilis* CotA and *Streptomyces coelicolor* EpoA were used as search templates, because both enzymes were successfully expressed in functional form in *E. coli* and oxidised the canonical laccase substrates ABTS (2′-azino-bis(3-ethylbenzthiazoline-6-sulphonic acid)) and 2,6-DMP (2,6-dimethoxyphenol)^23^,^24^. Only hits which contained the strictly conserved, consecutive copper binding motifs HXHG, HXH, HXXHXH and HCHXXXHXXXXM/L/F of multi copper oxidases were considered for cloning.

The genome sequence of *B. coagulans* contained a putative LMCO protein sequence annotated as “multicopper oxidase type 2” (NCBI reference sequence YP_004860005.1). The protein exhibited 59% amino acid sequence identity to the *B. subtilis* CotA search template. The genome sequence of *Gramella forsetii* KT0803 contained a putative LMCO protein sequence annotated as “blue copper oxidase” (NCBI reference sequence YP_861212.1). The protein exhibited 47.9% amino acid sequence identity to the *B. subtilis* CotA search template and contained a predicted TAT signal sequence with two probable cleavage sites after residues A20 and S33. The genome sequence of *Streptomyces pristinaespiralis* ATCC25486 contained a putative LMCO protein sequence annotated as “copper oxidase” (NCBI reference sequence ZP_06908025.1). The protein exhibited 71% amino acid sequence identity to the *S. coelicolor* A3 copper oxidase search template and contained a predicted TAT signal sequence with the first, most probable cleavage site after residue A41.

Once we found ABTS oxidising activity for *Gramella forsetii* LMCO, we used this sequence for a further round of Protein BLAST. The genome of *Marivirga tractuosa* DSM4126 contained a putative LMCO protein sequence annotated as “multicopper oxidase type 3” (NCBI reference sequence YP_004054188.1). The protein exhibited 54% amino acid sequence identity to the *G. forsetii* blue copper oxidase search template and contained a predicted TAT signal sequence with a probable cleavage site after position C32. A plasmid sequence pSLIN02 of *Spirosoma linguale* DSM74 contained a putative LMCO annotated as “bilirubin oxidase” (NCBI reference sequence YP_003391614.1). The protein exhibited 26.4% amino acid sequence identity to the *G. forsetii* blue copper oxidase search template and contained a predicted TAT signal sequence with a probable cleavage site after position G26. Although the *S. linguale* sequence contained all conserved copper binding motifs, it exhibited only 17% amino acid sequence identity to *B. subtilis* CotA.

Proteins with disulfide bridges are often difficult to express in the cytoplasm of *E. coli*. The crystal structure of *B. subtilis* CotA revealed that the folded, copper-loaded enzyme contains a disulfide bridge between residues Cys 229 and Cys 322^30^. We aligned *B. subtilis* CotA with other *Bacillus* LMCOs and observed that these residues are conserved in the previously characterised *B. pumilus* and *B. licheniformis* enzymes, while they were missing in the *B. clausii* and *B. coagulans* homologues (Supplementary Figure S1). The protein sequence of the recently characterised *B. clausii* LMCO^31^ was found to contain only one cysteine residue in addition to the strictly conserved copper ligand motif CotA_*B. subtilis*_ H_491_C_492_H_493_. The sequence of *B. coagulans* LMCO exclusively contained the copper ligand cysteine. Therefore, no disulfide bonds can be formed in *B. clausii* and *B. coagulans* LMCOs.

The putative LMCOs originated from highly divergent bacterial phyla, encompassing both Gram-positive and Gram-negative bacteria: *Firmicutes* (*B. coagulans*), *Actinobacteria* (*S. pristinaespiralis*) and *Bacteriodetes* (*G. forsetii*, *M. tractuosa*, *S. linguale*). The candidate genes were cloned into expression vectors for *E. coli* by PCR based methods or by gene synthesis (Table 1). In the case of *G. forsetti* and *S. pristinaespiralis* LMCOs, both full-length versions and N-terminally truncated versions lacking the predicted signal peptides were constructed.

### Activity yields and expression levels of phylogenetically diverse bacterial LMCOs in *E. coli*

Activity yields and expression levels of novel LMCOs were compared to previously described *B. subtilis, B. pumilus* and *B. clausii* CotA^23^,^28^,^31^. With the exception of *S. linguale* LMCO, moderate to high ABTS oxidising activity was observed after overnight induction (Table 2). In most cases a switch to static cultivation conditions after induction (imposing oxygen limitation) strongly increased volumetric activity (Table 2). The increase ranged from 1.4- to 118-fold. Only for *S. linguale* LMCO this effect was not observed, but here volumetric activities were generally very low. When cells were grown in fully aerated, baffled shake flasks under continuous, vigorous shaking, higher final optical densities were reached (Table 2). During late stages of oxygen-limited cultures with standard LB medium protein synthesis might be limited by restrictions in energy supply caused by a lack of fermentable carbohydrates or by a shift to acidic pH and organic acid accumulation due to mixed acid fermentation. Thus, we investigated whether the use of a medium with high buffer capacity and an additional fermentable carbon- and energy source (LBPG medium) facilitates increased yields of active LMCOs. A substantial increase in volumetric activity was achieved with this strategy in the case of *B. pumilus* and *B. subtilis* LMCOs; however, for the other enzymes a detrimental effect on activity yields was observed (Table 2).

Another factor influencing the yield of correctly folded proteins in *E. coli* are N-terminal signal peptides. The presence or absence of native N-terminal TAT signal sequences in the constructs used for expression of LMCOs influenced activity yields considerably (Table 2). A *G. forsetii* LMCO version beginning with the amino acid after the second predicted cleavage site (D34, pGoL3) yielded the highest volumetric activities (Table 2). A version starting with G21 (first predicted cleavage site, pGoL2) did not yield any active protein (data not shown). Activity yields for the full-length version of *G. forsetii* LMCO were reduced more than 50-fold (Table 2). Thus, the other *Bacteriodetes* LMCOs (*M. tractuosa, S. linguale*) were also expressed as versions lacking the predicted signal peptide, i.e. starting with residues corresponding to *G. forsetii* D34 (Table 1). The *M. tractuosa* construct facilitated volumetric activities in the same order of magnitude as pGoL3, while only very low activities were detected for *S. linguale* LMCO (Table 2). In the case of *S. pristinaespiralis*, ABTS oxidising activity was only detected for a construct which included the native TAT signal sequence. A version starting with A42 after the first predicted signal peptide cleavage site (pSpL2) did not yield any activity (data not shown). This finding is in contrast to a recent study of Gunne *et al.* who showed that a homologous enzyme of *Streptomyces sviceus* with 73.2% sequence identity to *S. pristinaespiralis* LMCO expressed well in *E. coli* without signal peptide^32^. The sequence of the *S. sviceus* signal peptide strongly differs from the *S. pristinaespiralis* signal peptide in the region before and after the predicted cutting site (data not shown). Possibly, *S. pristinaespiralis* LMCO could be expressed without signal peptide in *E. coli* if another starting residue is chosen, e.g. one after the second or third predicted cleavage site.

Heterologous DNA sequences encoding overexpressed genes often exhibit a different codon bias than that of the expression host. We investigated whether the co-expression of rare tRNAs has a positive effect on volumetric activities under the best expression conditions for each LMCO. Expression vectors were transformed into strains harboring the pRARE2 helper plasmid which supplies tRNAs for seven rare codons and has been shown to improve the expression levels of numerous human recombinant proteins in *E. coli*^17^. A significantly increased volumetric activity due to the helper plasmid was only achieved for *B. pumilus* and *B. clausii* LMCOs (Table 2). For the *B. subtilis*, *B. coagulans*, *S. pristinaespiralis* and *S. linguale* enzymes no significant effect was found. A detrimental effect of pRARE2 on volumetric activity was observed for *G. forsetii* and *M. tractuosa* LMCOs. Only the DNA sequence of *G. forsetii* was provided as codon optimised gene, containing only 4% rare codons. All other LMCOs were cloned as wild-type genes and contained 9-12% rare codons. The effect of pRARE2 on volumetric activity did not correlate with the percentage of rare codons in the DNA sequence.

**Figure 1:**
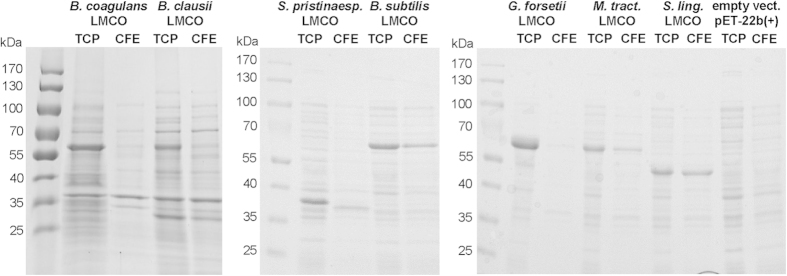
Expression levels of diverse bacterial LMCOs in *E. coli*. SDS PAGE of biomass-normalised (OD_600_) total cell protein (TCP) and cell free extract (CFE) samples prepared after overnight induction under static conditions. Samples were denatured for 20 min at 95 °C before loading and proteins were visualised by Coomassie Brilliant Blue staining. Expected molecular masses of recombinant LMCOs: *B. coagulans* 59.7 kDa, *B. clausii* 58.4 kDa, *S. pristinaespiralis* 32.6 kDa, *B. subtilis* 58.5 kDa, *G. forsetii* 59.0 kDa, *M. tractuosa* 59.6 kDa, *S. linguale* 51.1 kDa.

Protein expression levels were analysed by SDS-PAGE using samples of cultures with the highest volumetric activities. Specific bands of the expected size were detected in all cases in total cell protein (TCP) samples (Fig. 1). However, with the exception of *B. subtilis* and *S. linguale* LMCOs, the corresponding bands in cell free extracts (CFE) were much weaker, indicating that large fractions of the overexpressed proteins remained insoluble (Fig. 1). Solubility of the recombinant proteins did not correlate with activity yields, in particular with respect to *S. linguale* LMCO.

### Heat and storage stabilities

**Figure 2:**
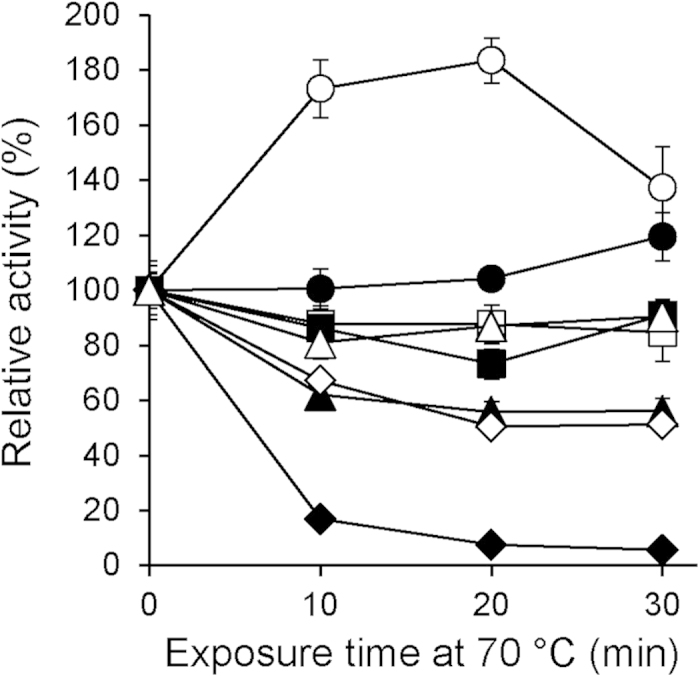
Heat stability of recombinant bacterial LMCOs and a fungal reference enzyme. Open circles: *B. clausii*, closed circles: *B. subtilis*, closed squares: *M. tractuosa*, open squares: *B. coagulans*, open triangles: *S. linguale*, closed triangles: *S. pristinaespiralis*, open diamonds: *G. forsetii*, closed diamonds: fungal laccase (*T. versicolor*). Average values and standard deviations of 3 replicate experiments.

Previously described bacterial *Bacillus* LMCOs have their highest activities at elevated temperatures and resist thermal denaturation^23^,^25^,^28^, a highly interesting property with respect to biotechnological applications. We compared the heat stability of phylogenetically diverse bacterial LMCOs to a commercially available fungal laccase. In agreement with previous reports^23^,^31^ all *Bacillus* LMCOs retained their full activity during 30 min incubation at 70 °C in cell-free extract (Fig. 2). The observed heat activation of *B. clausii* LMCO by a factor of two was also described recently by Brander *et al.*^31^. Two LMCOs derived from the Gram negative phylum *Bacteriodetes* (*S. linguale*, *M. tractuosa*) were also highly heat stable, retaining approximately 90% of their initial activity after 30 min exposure at 70 °C (Fig. 2). By contrast, a third Bacteriodetes LMCO (*G. forsetii*) lost 50% of the initial activity under the same conditions. Similar to previous reports on *Streptomyces griseus* and *Streptomyces coelicolor* LMCOs (actinomycetes)^24^,^33^, the homologous *S. pristinaespiralis* enzyme analysed in our study lost some activity, but was more heat stable than the fungal reference enzyme from *Trametes versicolor* (Fig. 2). The observed reduction of activity by 40% within 30 min at 70 °C matched data reported for *Streptomyces griseus* EpoA expressed in *E. coli*^24^.

**Figure 3:**
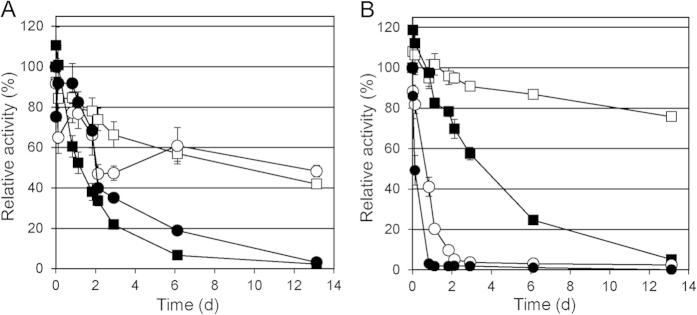
Storage stability of purified bacterial LMCOs in (**A**) 100 mM potassium phosphate buffer pH 7.0 and (**B**) double-distilled water. Open symbols: incubation at 25 °C, closed symbols: incubation at 45 °C; squares: *B. clausii* LMCO; circles: *B. coagulans* LMCO. Average values and standard deviations of relative activities (ABTS, pH 4, 30 °C) determined in 3 replicate experiments.

Technical use of enzymes requires high storage stabilities, preferably without cooling. Therefore, we analysed the stability of *B. coagulans* LMCO and *B. clausii* CotA in aqueous solution over prolonged periods of time with purified enzyme (LMCO purification: see below). In the case of *B. clausii* LMCO 40% of the initial activity was retained after 13 days of incubation at 25 °C in phosphate buffer with neutral pH (Fig. 3A). Inactivation at 45 °C proceeded faster; here 80% of the initial activity was lost within 3 days. The time course of activity loss of *B. coagulans* LMCO in phosphate buffer at 25 °C was similar; at 45 °C the enzyme exhibited slightly higher stability (Fig. 3A). Interestingly, storage stability of *B. clausii* LMCO was enhanced in double distilled water at both temperatures (Fig. 3B). By contrast, inactivation of *B. coagulans* LMCO in double distilled water proceeded faster than in phosphate buffer (Fig. 3B). Stabilities of bacterial LMCOs over a time period of weeks have rarely been studied by others. Brander *et al.* analysed the stability of *B. subtilis* and *B. clausii* LMCOs over a period of 25 h at 50 °C; at pH 8-10 they found 100% retention of activity 31. We observed a somewhat lower stability of *B. clausii* LMCO at 45 °C. Activity was reduced by 48% after 27 h of incubation at pH 7 in phosphate buffer. This might be due differences in the experimental setup (lower pH, different buffer) compared to Brandner *et al.*^31^. In double distilled water, the *B. clausii* enzyme retained 82% of the initial activity after 27 h at 45 °C. This value and a residual activity of 82% recorded for *B. coagulans* LMCO after 27 h of incubation at pH 7 in phosphate buffer matched the reported values more closely.

As expected, the analysed *Bacillus* LMCOs where highly stable when stored either at 4 °C in phosphate buffer or at -20 °C in 50% v/v glycerol; 60%-80% of the initial activities were left after 13 d both for the *B. clausii* and *B. coagulans* enzymes (data not shown).

### Substrate range, pH optimum and kinetic properties

One advantage of laccases and LMCOs is their broad substrate range. The screening of *B. subtilis*, *B. pumilus*, *S. pristinaespiralis*, *G. forsetii*, *M. tractuosa* and *S. linguale* LMCOs for oxidation of 91 potential laccase substrates has been described elsewhere^4^. In brief, *Bacillus* enzymes exhibited the broadest substrate range, while *G. forsetii* and *M. tractuosa* LMCOs oxidised fewer compounds and the number of transformed substrates was smallest for *S. pristinaespiralis* and *S. linguale* LMCOs. Here we provide similar data for the *B. clausii* and *B. coagulans* CotA-type LMCOs. The majority of *B. subtilis* CotA substrates were also oxidised by these enzymes (Table 3). Hence, promiscuity of the *B. clausii* and *B. coagulans* LMCOs was also higher than that of non-*Bacillus* bacterial LMCOs.

Purification of recombinant *Bacillus* LMCOs is simple because the majority of *E. coli* proteins can be separated by simple heat treatment^25^,^28^. We purified *B. clausii* and *B. coagulans* LMCOs by the same procedure as used for *B. pumilus* CotA. Purification tables and SDS-PAGE images of the consecutive purification steps are given in the Supplementary Materials (Figure S2, Table ST1). The remarkable resistance of *B. pumilus* CotA to denaturation at 95 °C in SDS sample buffer^28^ was also observed for *B. coagulans*, but not for *B. clausii*. In the case of *B. coagulans* LMCO, even 30 min of incubation in the presence of a strong detergent was not sufficient for complete denaturation (Supplementary Figure S2).

**Figure 4:**
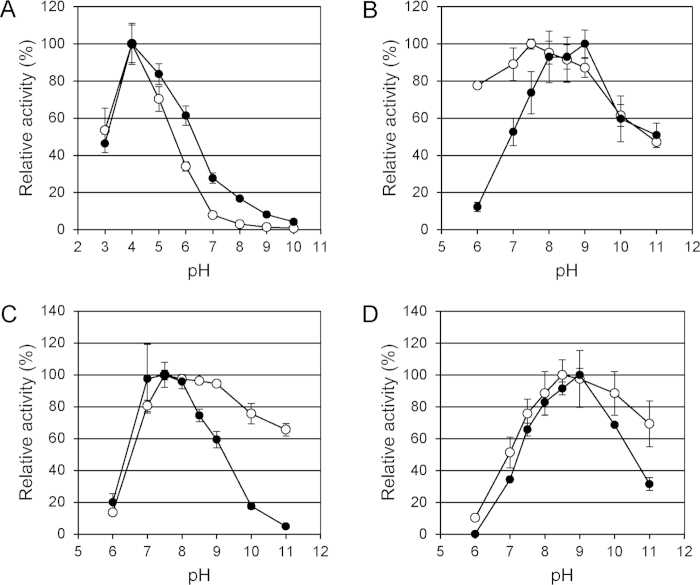
pH-activity profiles of *B. coagulans* (open symbols) and *B. clausii* (closed symbols) LMCOs. Substrates were (**A**) ABTS, (**B**) 2,6-dimethoxyphenol, (**C**) syringaldazine, and (**D**) guaiacol. Average values and standard deviations of 3 replicate experiments.

Fungal laccases are mostly active only at acidic pH^4^. Yet, some industrial processes require neutral to alkaline pH. The pH optimum of *B. subtilis, B. pumilus* and *B. licheniformis* CotA was reported to be 4-5 for the non-phenolic substrate ABTS and 7 for the phenolic substrates 2,6-DMP and syringaldazine (SGZ)^23^,^25^,^28^. As described elsewhere, the LMCOs derived from the Gram negative species *G. forsetii* and *M. tractuosa* also have a pH optimum of 4 for ABTS, while the typical phenolic laccase substrate 2,6-DMP is not oxidised at all^4^. The pH-activity profiles of the small EpoA-type LMCOs from the actinomycetes *S. coelicolor* and *S. griseus* were reported to be highly similar to *Bacillus* LMCOs in the case of ABTS, while a higher relative activity was reported for 2,6-DMP in the pH range of 8-10^24^,^33^. We confirmed these observations for the homologous *S. pristinaespiralis* enzyme elsewhere^4^. The only previously described CotA-type LMCO with high activity at pH 8-10 is the one from *B. clausii*^31^. We compared pH-activity profiles of the novel *B. coagulans* LMCO to *B. clausii* CotA for four different substrates. The optimum for ABTS oxidation was again pH 4 for both enzymes, with a continuous decline of relative activity towards neutral pH (Fig. 4A). Residual ABTS-oxidising activity of *B. clausii* at neutral pH was significantly increased compared to *B. coagulans* LMCO (Fig. 4A). In the case of 2,6-DMP, *B. coagulans* LMCO was as active as *B. clausii* LMCO at alkaline pH, but retained in addition high relative activity at pH 6-7 (Fig. 4B). In the pH range 8.5-11 *B. coagulans* LMCO outperformed *B. clausii* CotA when the phenolic compounds SGZ and guaiacol were used as substrates (Fig. 4C,D). Even at a rather extreme pH of 11, *B. coagulans* LMCO retained more than 60% of the activity recorded at the optimal pH. *B. coagulans* LMCO was highly stable at alkaline pH, even when 25% v/v dimethyl sulfoxide was added as co-solvent (Supplementary Figure S3). At room temperature, activity was completely retained for 5 days at pH10 and pH11. At 45 °C, 40-60% of the initial activity was left after the same incubation time.

Kinetic constants for purified *B. clausii* and *B. coagulans* LMCOs were determined with the two common laccase substrates ABTS and 2,6-DMP (Table 4). For each substrate, the experimentally defined, optimal pH was used (ABTS: *B.clausii* and *B. coagulans* pH 4.0; 2,6-DMP: *B. clausii* pH 8.0, *B. coagulans* pH 7.5). Michaelis-Menten plots are shown in the Supplementary Materials (Figures S4 and S5). Similar to data reported by Brander *et al.*^31^, both *B. clausii* and *B. coagulans* LMCO exhibited moderate, uncompetitive substrate inhibition when oxidising 2,6-DMP. Therefore, a modified Michaelis-Menten equation with a substrate inhibition term (*K*_*i*_) was used for curve fitting and calculation of kinetic constants in SIGMA-PLOT. Strong uncompetitive substrate inhibition was observed for *B. coagulans* LMCO with ABTS as substrate, while this effect was much less pronounced for the *B. clausii* enzyme (Figures S4 and S5). The *k*_cat_ and *K*_M_ values of *B. clausii* and *B. coagulans* LMCOs were in the same order of magnitude as values reported by others for fully copper-loaded *B. subtilis* CotA^27^, *B. licheniformis* CotA^25^ and *B. pumilus* CotA^28^. Kinetic parameters of *B. clausii* CotA with 2,6-DMP as substrate were determined previously by Brander *et al.*^31^. The values reported for conditions similar to ours (pH 7-8, *k*_cat_ 4.2-4.5 s^−1^, *K*_M_ 1500-2000 μM) were lower than our values. This could be due to differences in experimental procedures. In the case of SGZ it was not possible to fit a Michaelis-Menten model to the data; therefore, we did not calculate kinetic parameters (Supplementary Figures S4C and S5C). Most likely, the unusual activity profiles were due to substrate solubility problems^28^. The maximal specific activities for SGZ oxidation were 7.6 ± 0.5 μmol min^−1^ mg^−1^ for *B. clausii* LMCO and 145 ± 1.9 μmol min^−1^ mg^−1^ for *B. coagulans* LMCO at 0.125 and 2 mM substrate concentration, respectively.

## Discussion

The full potential of laccases and laccase-like multi-copper oxidases for sustainable production of chemicals and materials can only be realised if a broader set of robust and low-priced enzymes becomes available^34^. In particular with respect to activity at neutral to alkaline pH, resistance to heat denaturation and amenability to expression in simple bacterial expression systems, bacterial LMCOs bear high potential^35^. We expressed five novel LMCOs from diverse phyla of eubacteria in *E. coli* and directly compared them to previously described *Bacillus* LMCOs with respect to activity yields and biochemical properties. LMCOs derived from *Bacillus* species yielded the overall highest volumetric activities, followed by two enzymes from *Bacteriodetes*. In agreement with previous reports^27^,^28^, all LMCOs with volumetric activities above 100 U L^−1^ in *E. coli* shake flask cultures exhibited a strict requirement for static incubation (oxygen limitation) during the induction phase. Presumably, intracellular copper concentrations during aerobic growth of wild-type *E. coli* are generally too low for correct incorporation of all four metal ions in overexpressed LMCOs during folding. Thus, a static induction phase should always be used when characterising and screening novel and/or engineered LMCOs. The strict requirement for oxygen-limited conditions during induction hinders a scale-up to higher biomass concentrations. Oxygen saturation in high cell density fed-batch cultures of *E. coli* is usually kept above 10% in order to avoid a shift to fermentative growth^36^,^37^, as this would lead to rapid accumulation of toxic concentrations of organic acids. Induction of LMCO expression and shift to oxygen limitation very late in the process may solve the problem. If not, engineering of *E. coli* for increased intracellular copper concentrations under oxic conditions is necessary. One option is the co-expression of intracellular copper chaperones. Gunne *et al.* co-expressed the small soluble copper chaperone CopZ with *B. licheniformis* CotA which resulted in a 26% higher specific activity^38^.

No consistent effects of medium additives and co-expression of rare tRNAs were observed in our experiments. Thus, novel LMCOs should best be expressed initially with and without pRARE plasmids using several types of growth media. Volumetric activities of 2000-4000 U L^−1^ reached in our experiments under optimal conditions are among the highest reported for bacterial LMCOs. To our knowledge only one study reported values of a similar range: the maximum volumetric activity of *B. subtilis* CotA achieved with a static induction phase was 5614 U L^−1^ (ABTS, 37 °C)^27^. The maximal volumetric activity of another well-characterised LMCO in *E. coli*, wild-type *B. licheniformis* CotA was 410 U L^−1^ 39.

Our results indicate that bacterial LMCOs generally suffer from low solubility when overexpressed in *E. coli.* Solubility of proteins can be enhanced by substituting surface-exposed amino acids. The solubility of *B. licheniformis* CotA was substantially improved with a combined random and site-directed mutagenesis strategy, facilitating an 11-fold increased volumetric activity of 3400 U L^−1^ in *E. coli*^39^. A similar approach could be applied to *B. coagulans* and *G. forsetii* LMCOs which were characterised by a high amount of insoluble protein when overexpressed.

Impaired disulfide bond formation in the cytoplasm of *E. coli* does not seem to be related to misfolding and aggregation. *B. clausii* and *B. coagulans* CotA lack cysteine residues which can form disulfide bonds; however, activity yields were not higher than that of the *B. subtilis* homologue for which a disulfide bond has been observed in the crystal structure^30^.

Stability of enzymes is a key factor for their technical applicability. The high resistance of *Bacillus* LMCOs to thermal and chemical and inactivation was first described for *B. subtilis* CotA by Martins *et al.*^23^ and was since then observed for several homologous enzymes derived from, e.g., *B. licheniformis, B. pumilus* and *B. clausii*^25^,^28^,^31^. Although the LMCOs derived from Gram negative bacteria and actinomycetes in our study also exhibited higher heat stability than *Trametes versicolor* laccase, the *Bacillus* enzymes were clearly the most stable ones. In addition we demonstrate that *B. clausii* and *B. coagulans* LMCO solutions can be stored for weeks at ambient conditions with activity retention of 50-75%, indicating that cooling is not necessary. It is likely that storage stability can be further increased by adding stabilisers such as sucrose.

LMCOs that are active at alkaline pH increase the range of applications for this class of enzymes, e.g. for lignin modification. The novel LMCO derived from *B. coagulans* exhibited an astonishingly high relative activity at strongly alkaline pH. With respect to the substrates SGZ and guaiacol, more alkaline pH conditions were tolerated compared to previously described alkaliphilic *B. clausii* CotA^31^. To our knowledge strong activity at pH 11 has not been reported before for any laccase or LMCO.

Other key factors determining the suitability of an enzyme for biotechnological processes are catalytic efficiency and substrate range. Maximal turnover numbers (*k*_cat,_ derived from maximal specific activity) and Michaelis constants (*K*_M_, half saturation constant) of *B. coagulans* LMCO recorded for ABTS and 2,6-DMP were in the same order of magnitude as those of previously characterised *Bacillus* LMCOs^25^,^27^,^28^,^31^. Similar to previously analysed *B. subtilis* and *B. pumilus* LMCOs^4^, the homologous enzymes derived from *B. coagulans* and *B. clausii* were able to oxidize a very broad range of (mostly phenolic) compounds. This is in contrast to the rather limited substrate range of LMCOs derived from Gram-negative bacteria and Actinomycetes that cannot oxidize many of the typical phenolic substrates of fungal laccases, e.g. guaiacol and acetosyringone^4^. We suggest that such compounds, yielding coloured products upon oxidation, should be used if one wants to specifically screen for bacterial LMCOs with a broad substrate range.

In summary, this study confirms that *Bacillus* LMCOs offer a variety of beneficial properties, not matched by related enzymes derived from other bacterial species. Due to the high expression levels and activity yields in *E. coli*, high activity at alkaline pH, a broad substrate range and high stability, *B. coagulans* CotA seems to be a promising candidate for further development.

## Methods

### Bacterial strains

*Bacillus clausii* DSM8716, *Bacillus coagulans* DSM1, *Marivirga tractuosa* DSM4126, *Spirosoma linguale* DSM74 and *Streptomyces pristinaespiralis* DSM40338 were obtained from the German collection of microorganisms and cultivated according to the instructions of the supplier (DSMZ, http://www.dsmz.de). *E. coli* strain JM109 [genotype *endA*1 *recA*1, *gyrA*96*, thi*, *hsdR*17,(rK^−^, mK^+^), *relA*1, *supE*44, l-, Δ(*lac-proAB*), (F’, *traD*36, *proAB*, *lacI*^q^*Z*ΔM15)] was purchased from Promega (Madison, USA). *E. coli* BL21(DE3) was purchased from Agilent Technologies. Rosetta™ 2(DE3) [genotype F– *ompT hsdS*_B_(rB^–^ mB^–^) *gal dcm* (DE3) pRARE2 (Cam^R^)] was purchased from Merck Millipore (Germany).

*E. coli* strains were routinely grown in LB medium at 37 °C and 150 rpm. For plasmid selection 100 mg L^−1^ ampicillin was added to LB agar plates and liquid medium and 34 μg/mL chloramphenicol when cells were co-transformed with pRARE2.

### Database search and construction of plasmids

Genes encoding putative LMCOs were identified in bacterial genomes by Protein BLAST searches (http://blast.ncbi.nlm.nih.gov/Blast.cgi) using sequences of the following bacterial proteins with confirmed ABTS oxidising activity as search templates: *B. subtilis* CotA^23^ (NCBI reference sequence NP_388511.1), *S. coelicolor* A3 copper oxidase^33^ (NCBI reference sequence NP_630785.1) and *G. forsetii* blue copper oxidase (NCBI reference sequence YP_861212.1, this study). The presence of putative N-terminal signal sequences was analysed with the Signal P 3.0-HMM program using appropriate networks for the analysed organisms (http://www.cbs.dtu.dk/services/SignalP/)^40^. Alignments of amino acid sequences and calculations of amino acid sequence similarities were carried out with the MegAlign^TM^ software (LASERGENE, Madison, USA) using the ClustalW algorithm.

Plasmids used in this study for expression of bacterial LMCOs are listed in Table 1. Primer sequences and details of plasmid construction are described in the Supplementary Material. Genomic and plasmid DNA was extracted with commercial kits according to the instructions of the suppliers. Gene sequences, cloning sites and upstream and downstream regions at the 5′ and 3′ ends of LMCO-encoding inserts were controlled by sequencing. The nucleotide sequences of all genes cloned from genomic DNA matched the corresponding database sequences to 100%. *E. coli* BL21(DE3) was used as host strain for pET derived plasmids. *E. coli* JM109 (overexpressing *lacI*^*q*^ from the genome) was used as host strain for pQE-60 derived plasmids. In order to test the effect of additional copies of rare tRNAs, *E. coli* Rosetta 2 (DE3) competent cells (Novagen) harboring pRARE2 were transformed with pGoL3, pSiL, pMtraL, pBuL, pLOM10, pBoL and pBaL. JM109 competent cells were transformed with pRARE2, previously isolated from Rosetta 2 cells, made competent and then transformed with pSpL3.

### Expression conditions and cell lysis

For expression of recombinant bacterial LMCOs either LB (5 g L^−1^ yeast extract, 10 g L^−1^ tryptone, 5 g L^−1^ NaCl) or LBPG (phosphate buffered, LB-like medium with glucose: 5 g L^−1^ yeast extract, 10 g L^−1^ tryptone, 7.25 g L^−1^ Na_2_HPO_4_ 2H_2_O, 3 g L^−1^ KH_2_PO_4_, 4 g L^−1^ glucose, pH 7.2) medium was used. Expression studies were carried out in n = 3 biological replicates per condition. Shake flask cultures were inoculated 1:50 from individual overnight tube pre-cultures (37 °C, 160 rpm) and incubated at 30 °C and 160 rpm until an OD_600_ of 0.4-0.5. Subsequently, the cultivation temperature was reduced to 25 °C, the expression of recombinant proteins was induced by the addition of IPTG 1 mM and CuCl_2_ 0.25 mM final concentration, respectively. A subset of experiments was carried out in baffled flasks (total volume 500 mL, liquid volume 60 mL) which were continuously shaken at 160 rpm until the end of the experiment (SF shaken, aerobic growth). Another subset of experiments was carried out in normal Erlenmeyer flasks (total volume 200-300 mL, liquid volume 60 mL) which were shifted from 160 to 100 rpm agitation at induction and to 0 rpm (static incubation) 4 h after induction (SF static). LBPG cultures were shifted to incubation without agitation directly after induction. Static incubation leading to oxygen-limited growth had been shown previously to increase the yield of fully copper loaded bacterial LMCO in *E. coli*^27^. Final optical densities were determined at 600 nm with a microplate reader after appropriate dilution of samples. Volumetric activities were determined with OD_600_-normalised, lysed cell suspensions without centrifugation. Cells were harvested after overnight incubation by centrifugation (1 min, ≥12000 x g) and cell pellets were frozen at –80 °C. Pellets were thawed and resuspended to a calculated OD_600_ of 10 in 1x CellLytic B solution (Sigma-Aldrich) containing 0.2 mg mL^−1^ lysozyme and 0.5 μL mL^−1^ Benzonase® Nuclease (Novagen). Cell lysis was carried out for 20 min at room temperature and 200 rpm. LMCO activity was quantified with the ABTS assay (see below) except for *S. linguale* LMCO where activity was determined with 0.5 mM 2,6-dimethoxyphenol (2,6-DMP) at pH 7.0. Expression conditions with the highest activity yields were used for experiments with the pRARE2 helper plasmid supplying rare tRNAs.

For heat stability experiments and SDS-PAGE analysis, cell debris was removed from the lysed samples by centrifugation (5 min, ≥12000 x g) to recover cell free extract (CFE). Before loading on SDS-PAGE gels, CFE samples were mixed 1:1 with 2x reducing SDS-PAGE sample buffer. Total cell protein (TCP) samples for SDS-PAGE analysis were prepared by resuspending cells to an OD_600_ of 10 in 1x SDS-PAGE sample buffer. All SDS-PAGE samples were denatured at 95 °C for 20 min before electrophoresis.

### Biochemical assays

Enzymatic activity of lysed cells and CFE was determined at room temperature with 0.5 mM ABTS as substrate in 50 mM tartaric acid buffer at pH 4^41^ in transparent polystyrene 96-well microplates (Nunc). The reactions were followed with a microplate reader (Bio Tek Synergy Mx spectrophotometer) at 420 nm. Enzymatic activity was calculated using an extinction coefficient for mono-oxidised ABTS of 36′000 M^−1^ cm^−1^ 41. Resistance to heat denaturation was determined by incubating 100 μL CFE aliquots in 1.5 mL Eppendorf tubes at 70 °C and 300 rpm in a Thermomixer (Eppendorf). Samples were periodically taken and directly assayed at room temperature for enzymatic activity (ABTS, pH 4, *S. linguale*: 2,6-DMP, pH 7). Commercial *Trametes versicolor* laccase (Sigma-Aldrich) was dissolved to a concentration of 0.05 mg/mL in the same buffer as used for cell lysis (1 x CellLytic B). *B. clausii* and *B. coagulans* LMCOs were purified from overnight shake flask cultures with static induction phase as described previously^28^. Briefly, cell free extracts were prepared from 0.7 L induced overnight cultures by a combined freeze-thaw, lysozyme and sonication procedure. Non-heat stable proteins were precipitated by incubation at 70 °C for 20 min. LMCOs were further separated from contaminating proteins by anion exchange chromatography and size exclusion chromatography, which was sufficient for achieving protein samples of high purity as determined by SDS-PAGE. Protein concentrations after each purification step were determined with the Bradford method using premixed reagent (Sigma). Purified LMCOs were quantified at 280 nm with a Nanodrop device (Thermo Fisher Scientific, Waltham, USA) using extinction coefficients calculated from protein sequences (http://web.expasy.org/protparam/). Storage stabilities of purified enzyme preparations were determined after dilution in the respective storage solutions to initial volumetric activities of 0.3-0.4 U mL^−1^ (ABTS, pH 4, 30 °C). Samples were incubated at 25 °C and 45 °C and aliquots were assayed at regular intervals for ABTS oxidising activity at 30 °C.

The substrate range was analysed in 96-well plates by monitoring spectroscopic changes as described previously^4^,^28^. Wells contained 1 mM of each substrate in McIlvaine buffer (0.1-0.2 M citrate-phosphate)^42^ with pH 6 and reactions were initiated by addition of CFE. Compounds were counted as substrate when the UV/Vis spectrum was significantly changed after 24 h of incubation at 37 °C compared to control wells with CFE of a strain carrying the empty expression vector pET22b.

The relative activity at different pH values was recorded at 30 °C using Teorell-Stenhagen universal buffer because of its wide pH range of 2-12^43^. Substrates were added at concentrations that yielded the highest specific activities during determination of kinetic constants. At high pH values auto-oxidation was observed in enzyme free controls for the substrates 2,6-DMP and guaiacol. In these cases, auto-oxidation rates were subtracted from rates measured in experiments with enzymes before calculating relative activities.

Kinetic constants of *B. clausii* and *B. coagulans* LMCOs were determined at 30 °C with purified enzyme preparations as described by Reiss *et al.*^28^, except that a Michaelis-Menten equation with uncompetitive substrate inhibition term was used for parameter estimation with SIGMA-PLOT (Systat Software Inc., San Jose, USA): *v = v*_max_*[*S*]/(*K*_M_ + [S]*(1 + [*S*]/*K*_i_)). Experiments were performed at the optimal pH for ABTS (pH 4) and 2,6-DMP (pH 7.5-8-0) in McIlvaine buffer.

## Additional Information

**How to cite this article**: Ihssen, J. *et al.* Biochemical properties and yields of diverse bacterial laccase-like multicopper oxidases expressed in *Escherichia coli.* *Sci. Rep.* **5**, 10465; doi: 10.1038/srep10465 (2015).

## Supplementary Material

Supporting Information

## Figures and Tables

**Table 1:** Plasmids used in this study.

**Plasmid**	**Description**	**Marker**	**Reference**
pQE-60	Medium copy number vector for IPTG-inducible expression, P_tac_, ori: pBR322	Amp^R^	Qiagen
pET-22b(+)	Medium copy number vector for T7 polymerase dependent, IPTG-inducible expression, *lacI,* P_T7_, ori: pBR322	Amp^R^	Novagen
pJET/Blunt	Multi-purpose cloning vector	Amp^R^	Fermentas
pBpL6	*B. pumilus* CotA laccase in pQE-60	Amp^R^	28
pBuL	*B. pumilus* CotA laccase in pET-22b(+)	Amp^R^	This study
pLOM10	*B. subtilis* CotA laccase in T7 polymerase dependent, IPTG-inducible expression vector pET-21a	Amp^R^	23
pBaL	*B. clausii* CotA laccase in pET-22b(+)	Amp^R^	This study
pBoL	*B. coagulans* CotA laccase in pET-22b(+)	Amp^R^	This study
pGoL1	codon-optimised *G. forsetii* MCO in pET-22b(+)	Amp^R^	This study
pGoL2	codon-optimised *G. forsetii* MCO G_21_-V_559_ (without signal sequence, predicted cleavage site 1) in pET-22b(+)	Amp^R^	This study
pGoL3	codon-optimised *G. forsetii* MCO D_34_-V_559_ (without signal sequence, predicted cleavage site 2) in pET-22b(+)	Amp^R^	This study
pMtraL	*M. tractuosa* MCO D_32_-V_565_ (without predicted signal sequence) in pET-22b(+)	Amp^R^	This study
pSpL1	full-length *S. pristinaespiralis* MCO in pJET/Blunt	Amp^R^	This study
pSpL2	*S. pristinaespiralis* MCO A_42_-H_338_ (without predicted signal sequence) in pQE-60	Amp^R^	This study
pSpL3	full-length *S. pristinaespiralis* MCO in pQE-60	Amp^R^	This study
pSiL	*S. linguale* MCO C_27_-N_496_ (without predicted signal sequence) in pET-22b(+)	Amp^R^	This study
pRARE2	Supplies tRNAs for seven rare codons in *E. coli*: AUA, AGG, AGA, CUA, CCC, GGA, and CGG, all under the control of their native promoter	Cam^R^	Novagen

**Table 2:** Final optical densities and volumetric activity yields in shake flask cultures (SF) of *E. coli* expressing bacterial LMCOs.

**Enzyme**	**Plasmid**	**Final optical density (600 nm)±S.D.**				**Volumetric activity [μmol min**^−**1**^ **L**^−**1**^**]±S.D.**			
		**LB**		**LBPG**	**+pRARE2**	**LB**		**LBPG**	**+pRARE2**
		**SF shaken**	**SF static**	**SF static**	**best cond.**	**SF shaken**	**SF static**	**SF static**	**best cond.**
*B. pumilus* CotA	pBuL	3.8 (±0.2)	1.2 (±0.1)	1.0 (±0.11)	1.7 (±0.6)	12 (±1)	97 (±19)	617 (±81)	4157 (±452)
*B. subtilis* CotA	pLOM10	4.5 (±0.2)	1.5 (±0.1)	2.4 (±1.0)	1.2 (±0.1)	18 (±1)	526 (±47)	2648 (±273)	2612 (±217)
*B. clausii* CotA	pBaL	2.3 (±0.4)	1.5 (±0.1)	1.2 (±0.1)	1.5 (±0.06)	8.6 (±1.4)	60 (±9)	19 (±2)	110 (±8)
*B. coagulans* CotA	pBoL	2.4 (±0.4)	1.3 (±0.4)	2.0 (±0.1)	1.3 (±0.04)	7.8 (±0.7)	924 (±133)	52 (±5)	719 (±25)
*G. forsetii* LMCO with SP	pGoL1	n.a.	2.7 (±0.4)	2.0 (±0.4)	n.a.	n.a.	36 (±8)	26 (±5)	n.a.
*G. forsetii* LMCO w/o SP	pGoL3	5.4 (±0.2)	2.5 (±0.2)	1.3 (±0.2)	1.9 (±0.1)	137 (±12)	2440 (±244)	220 (±32)	422 (±3)
*M. tractuosa* LMCO w/o SP	pMtraL	4.3 (±0.1)	1.7 (±0.1)	1.1 (±0.3)	1.3 (±0.04)	30 (±5)	1615 (±118)	1356 (±128)	353 (±47)
*S. pristinaesp.* LMCO with SP	pSpL3	6.6 (±0.5)	1.7 (±0.1)	2.3 (±0.2)	1.2 (±0.1)	57 (±14)	83 (±7)	2.7 (±0.7)	85.5 (±5.7)
*S. linguale* LMCO w/o SP	pSiL	5.2 (±0.2)	1.7 (±0.1)	1.9 (±0.2)	1.4 (±0.2)	3.0 (±0.1)	2.1 (±0.6)	2.4 (±1.6)	5.1 (±0.9)
w/o=without, SP=signal peptide, S.D.=standard deviation, n.a.=not analysed									

**Table 3:** Substrate range of *B. clausii* and *B. coagulans* LMCOs compared to previously characterised *B. subtilis* CotA (Reiss *et al.* 4). Out of 91 tested compounds only those are shown which were oxidised by at least one of the three enzymes.

**Structural group**	**Compound**	***B. subt.***^4^	***B. clau.***	***B. coag.***
Aromatic carboxylic acids	*p*-Coumaric acid	+/−	+	+
	Caffeic acid	+	−	−
	Ferulic acid	+	+/−	+/−
	Sinapic acid	+	+	+
	3,4-Dihydroxybenzoic acid	+	+/−	+/−
	Gallic acid	+	−	−
	Syringic acid	+	+	+
	3-Amino-4 hydroxybenzoic acid	+	−	+/−
	4-Amino-3 hydroxybenzoic acid	+	+	+
	Vanillic acid	+/−	+/−	+/−
	3-(Dimethylamino) benzoic acid	−	+/−	+
	3-Hydroxyanthranilic acid	+	−	−
	*p*-Hydroxyphenylpyruvic acid	+	−	−
Aromatic alcohols	Vanillyl alcohol	+	+	+
	Isovanillyl alcohol	+	+	+/−
	Coniferyl alcohol	+	+	+
	Tyrosol	+	−	−
	*p*-Cresol	+	+/−	−
	2,6-Dimethylphenol	+	−	+
	Catechol	+	+	+
	4-Methylcatechol	+	+	+
	Pyrogallol	+	+/−	+/−
	2,6-Dimethoxyphenol	+	+	+
	3,4,5-Trimethoxyphenol	+/−	−	−
	Guaiacol	+	+	+
	Hydroquinone	+	+	+
	Mesitol	+	+	+/−
	3-Methylcatechol	+	+	+
	Eugenol	+	+	+
	Arbutin	+	+	+
	Resveratrol	+	+/−	+/−
	Quercetin hydrate	+/−	+	+
Aromatic ketones	Acetovanillone	+	+/−	+
	Acetosyringone	+	+/−	+/−
Aromatic aldehydes	*o*-Vanillin	+	+	+
	Syringaldehyde	+	+/−	+/−
	Ethyl vanillin	+	+	+
	Vanillin	+/−	−	+/−
	Sinapaldehyde	+	+	+
	Coniferyl aldehyde	+	+	+
Aromatic amines	Dopamine hydrochloride	+	+	+
Aromatic esters	Methyl vanillate	+	+/−	+/−
	Methylsyringate	+	−	−
Aromatic amides	Syringamide	+	+	+
	*N*-Hydroxyacetanilide	+	+	+
Polyphenol	Tannic acid	+	+	+
N-heterocycles	Violuric acid hydrate	−	+/−	+/−
	1-(3-Sulfophenyl)-3-methyl-2-pyrazolin-5-one	+	+	+
	1-(4-Sulfophenyl)-3-methyl-5-pyrazolone	+	+	+
Aromatic azo compounds	ABTS	+	+	+
	Syringaldazine	+	+	+
Triphenyl compounds	Cresol red sodium salt	+	+	+
Chromans	(+)-Catechin hydrate	+	+	+
	(−)-Epicatechin	+	+	+
Phenothiazines	Phenothiazine	+	+	+
	Promazine hydrochloride	+	+	+
Benzonitriles	2,3-Dimethoxybenzonitrile	+/−	−	−
Naphtalenes	1-Nitroso-2-naphthol-3,6-disulfonic acid	+	+	+
	2-Nitroso-1-naphthol-4-sulfonic acid	+	+	+
	1-Amino-2-naphthol-4-sulfonic acid	+	−	+/−

**Table 4:** Kinetic constants of *B. coagulans* and *B. clausii* LMCOs compared to previously characterised *B. pumilus* CotA^28^. Enzyme assays were carried out at the optimal pH for each substrate. Parameters were estimated with a Michaelis Menten equation with uncompetitive substrate inhibition.

		***B. coagulans***	***B. clausii***	***B. pumilus***^28^
ABTS	*K*_M_ [μM]	31 ±3	132 ±11	80 ±4
	*k*_cat_ [s^−1^]	69 ±3.1	90 ±2.3	291 ±2.7
	*K*_i_ [mM]	0.94 ±0.11	44 ±2.3	−
2,6-DMP	*K*_M_ [μM]	628 ±67	8535 ±2394	680 ±27
	*k*_cat_ [s^−1^]	17 ±0.8	65 ±15.2	11 ±0.1
	*K*_i_ [mM]	36 ±9.8	3.8 ±1.2	−
