# Supplementary material for: Biochemical properties and yields of diverse bacterial laccase-like multicopper oxidases expressed in Escherichia coli
Source: Sci Rep. 2015 Jun 12;5:10465. doi: 10.1038/srep10465 (PMC4464401; doi:10.1038/srep10465)
Supplement: Supporting Information [file srep10465-s1.doc]

Supplementary Material

Biochemical properties and yields of diverse bacterial laccase-like multicopper oxidases expressed in *Escherichia coli*

Julian Ihssen*^#^, Renate Reiss*, Ronny Luchsinger, Linda Thöny-Meyer and Michael Richter

* equal contribution

^#^ corresponding author, e-mail: julian.ihssen[@empa.ch](mailto:michael.richter@empa.ch)

Empa, Swiss Federal Laboratories for Materials Science and Technology,

Laboratory for Biointerfaces, Lerchenfeldstr.5, 9014 St. Gallen, Switzerland

Supplementary figures and tables

**
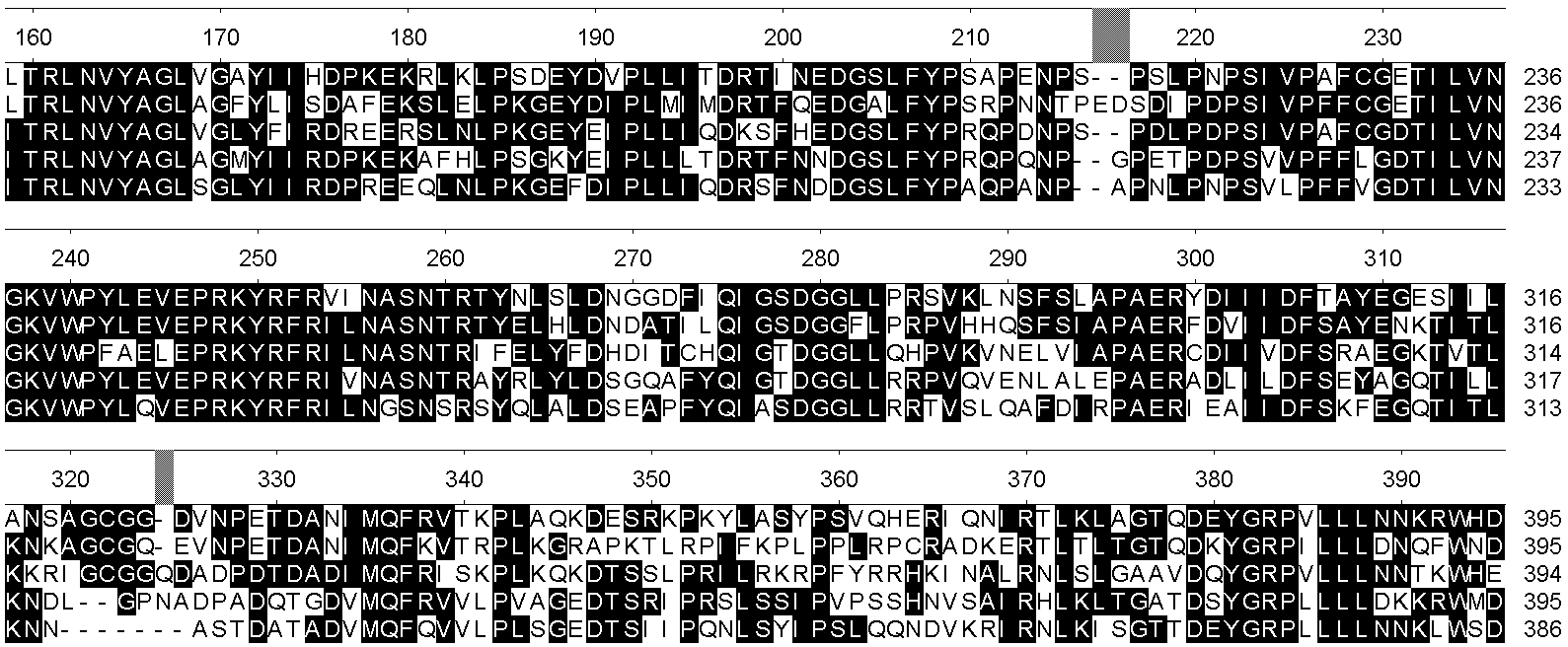
**

*B. subtilis
B. pumilus
B. licheniformis
B. coagulans
B. clausii*

*B. subtilis
B. pumilus
B. licheniformis
B. coagulans
B. clausii*

*B. subtilis
B. pumilus
B. licheniformis
B. coagulans
B. clausii*

**Supplementary Figure S1**. Alignment of amino acid sequences of *Bacillus* CotA type LMCOs in the region with disulfide bridge-forming cysteines (marked with arrows). Amino acid numbering according to the *B. subtilis* sequence is derived from the crystal structure 1GSK.

**
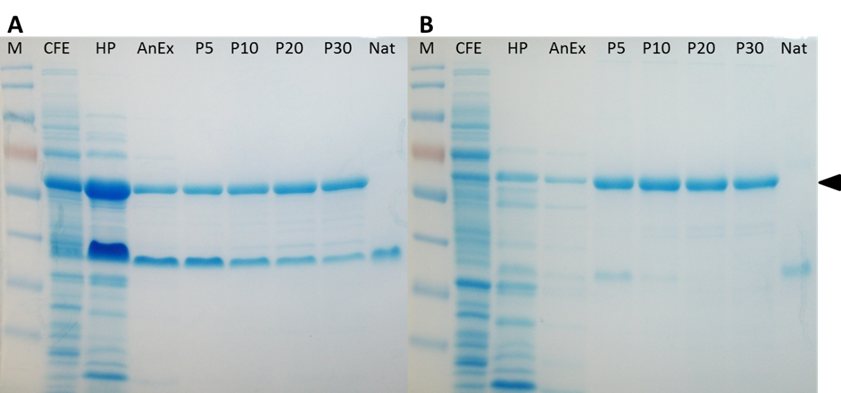
**

kDa

170

130

100

70

55

40

35

25

**Supplementary Figure S2**. SDS-PAGE analysis of (A) *B. coagulans* CotA and (B) *B. clausii* CotA after consecutive purification steps. CFE: cell free extract, HP: after heat purification (20 min 70 °C), AnEx: after anion exchange chromatography, P: purified protein after gel filtration; numbers indicate the time in minutes used for denaturation of proteins at 95 °C in SDS-sample buffer, Nat: native protein sample after gel filtration (prepared with SDS- and mercaptoethanol-free loading buffer, no heat denaturation). Arrow: Size corresponding to theoretical molecular mass (B*. coagulans*: 59.7 kDa, *B. clausii*: 58.4 kDa).

| Supplementary Table ST1 l Purity of *B. coagulans* (Bco) and *B. clausii* (Bcl) LMCOs after subsequent purification steps | | | | |
| --- | --- | --- | --- | --- |
| Sample | Total activity [U] | Specific activity [U/mg] | Yield [%] | Purification factor |
| Bco cell free extract | 283 | 1.0 | 100 | 1.0 |
| Bco heat purified | 228 | 4.9 | 81 | 4.9 |
| Bco anion exchange | 164 | 11 | 58 | 11 |
| Bco gel filtration | 124 | 28 | 44 | 28 |
| Bcl cell free extract | 168 | 0.3 | 100 | 1.0 |
| Bcl heat purified | 114 | 4.1 | 68 | 14 |
| Bcl anion exchange | 88 | 9.6 | 52 | 32 |
| Bcl gel filtration | 39 | 24 | 23 | 80 |


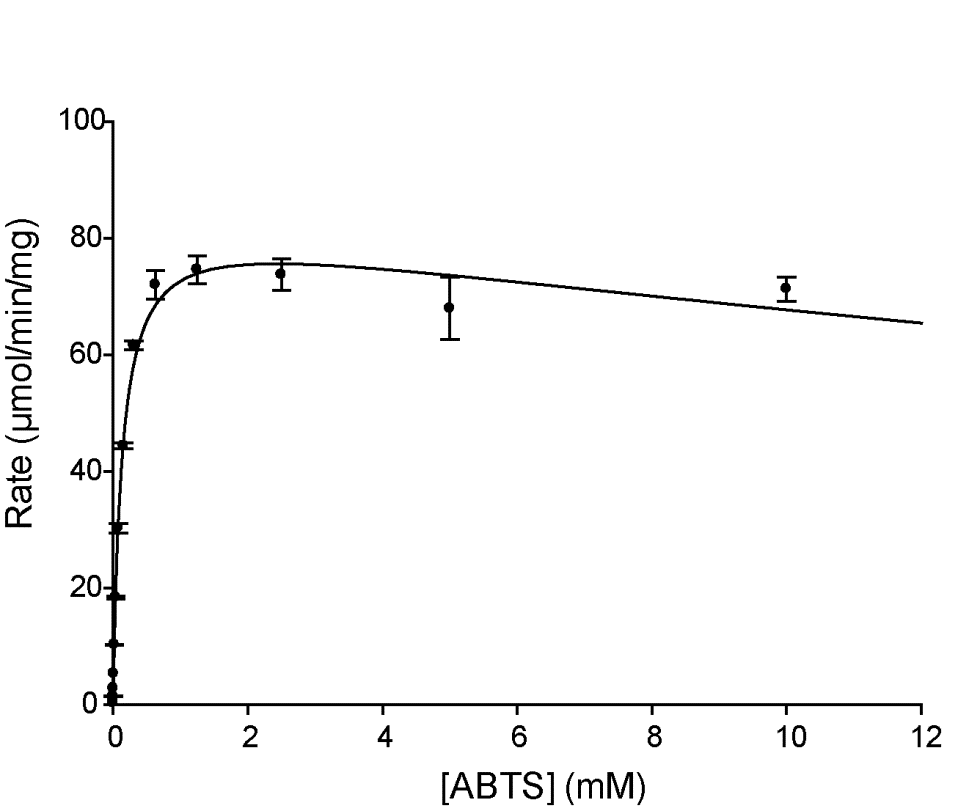


A

Value ±Std. Error 95% Conf. Interval

*V*_max_ 92.59 2.423 87.70 to 97.48

*K*_M_ 0.1319 0.0112 0.109 to 0.154

*K*_i_ 44.41 10.85 22.49 to 66.32


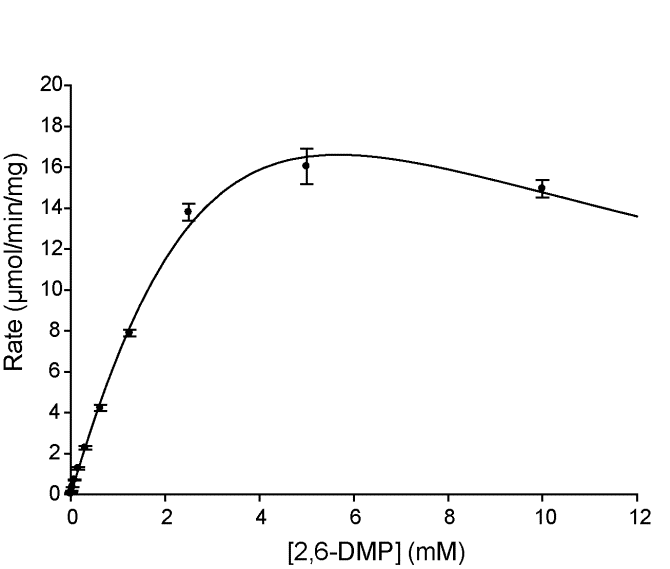


B

Value ±Std. Error 95% Conf. Interval

*V*_max_ 66.62 15.64 35.00 to 98.24

*K*_M_ 8.535 2.394 3.695 to 13.38

*K*_i_ 3.764 1.219 22.49 to 66.32


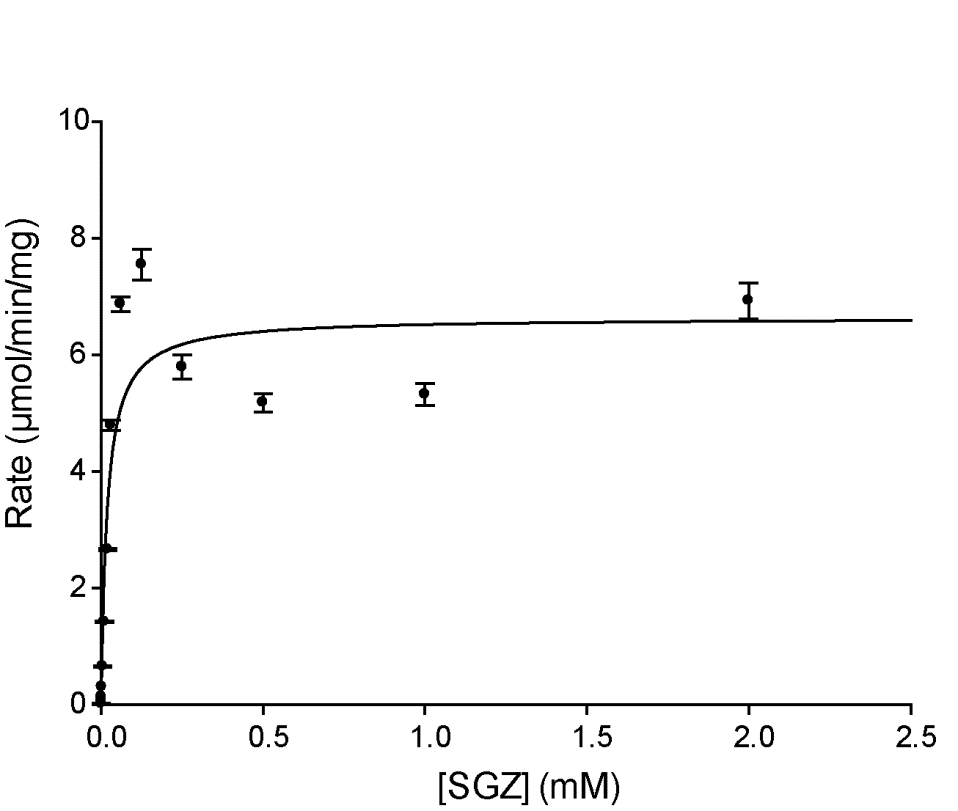


C

**Supplementary Figure S3**. Specific activity of *B. clausii* LMCO at 30°C in dependency of substrate concentration. (A) ABTS, pH 4.0, 2.7 µg mL^-1^ protein; (B) 2,6-DMP, pH 8.0, 27 µg mL^-1^ protein; (C) SGZ, pH 7.5, 24 µg mL^-1^ protein. Dots and error bars: average values and standard deviations of 3 replicate experiments. Solid line: Michaelis-Menten model with substrate inhibition fitted to experimental data with the least squares method in SIGMA-PLOT. Kinetic parameters in graphs were derived from fitted models.


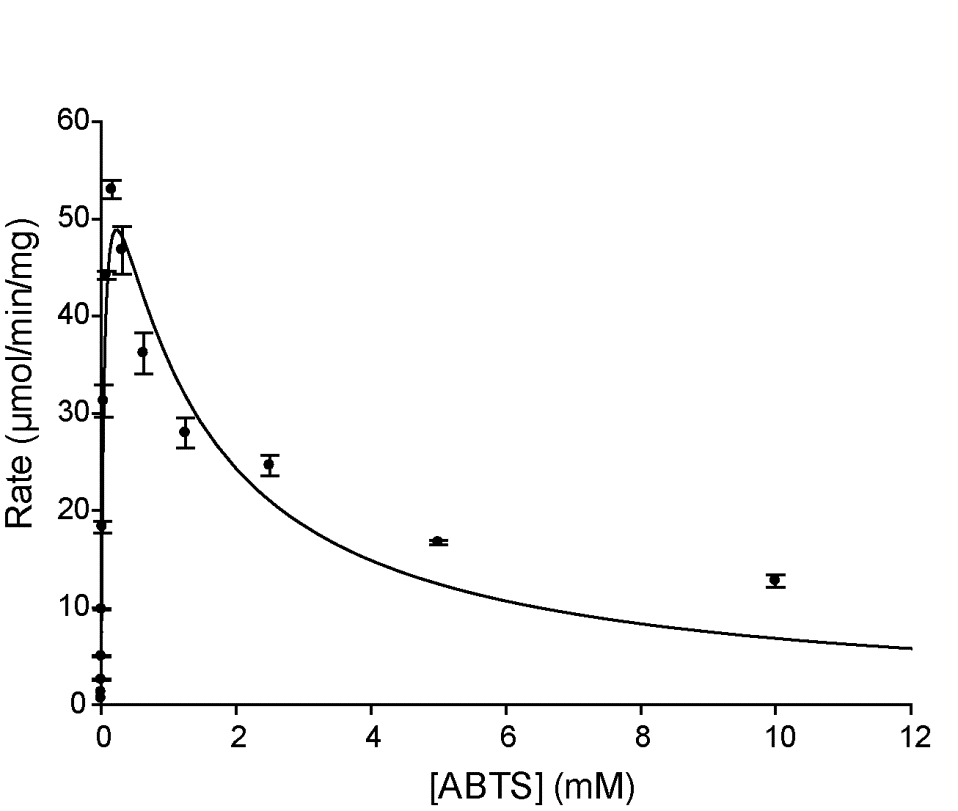


A

Value ±Std. Error 95% Conf. Interval

*V*_max_ 68.88 3.091 62.64 to 75.12

*K*_M_ 0.0307 0.0035 0.0237 to 0.0378

*K*_i_ 0.949 0.109 0.730 to 1.168


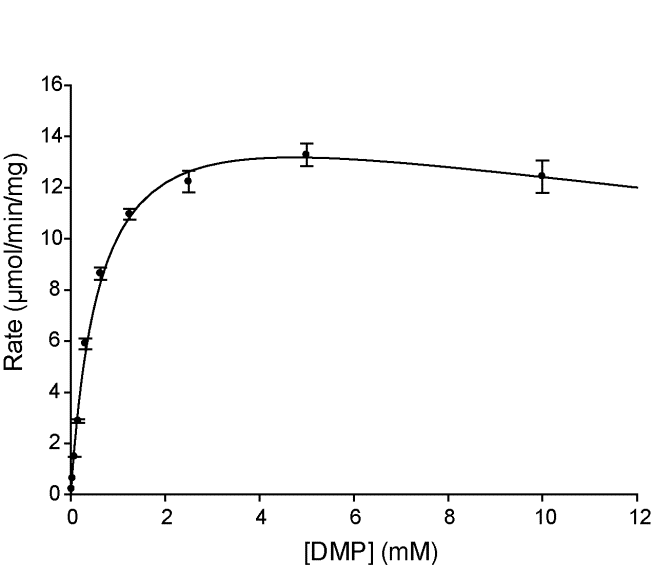


B

Value ±Std. Error 95% Conf. Interval

*V*_max_ 16.69 0.789 15.06 to 18.31

*K*_M_ 0.628 0.067 0.491 to 0.765

*K*_i_ 35.52 9.845 15.32 to 55.72


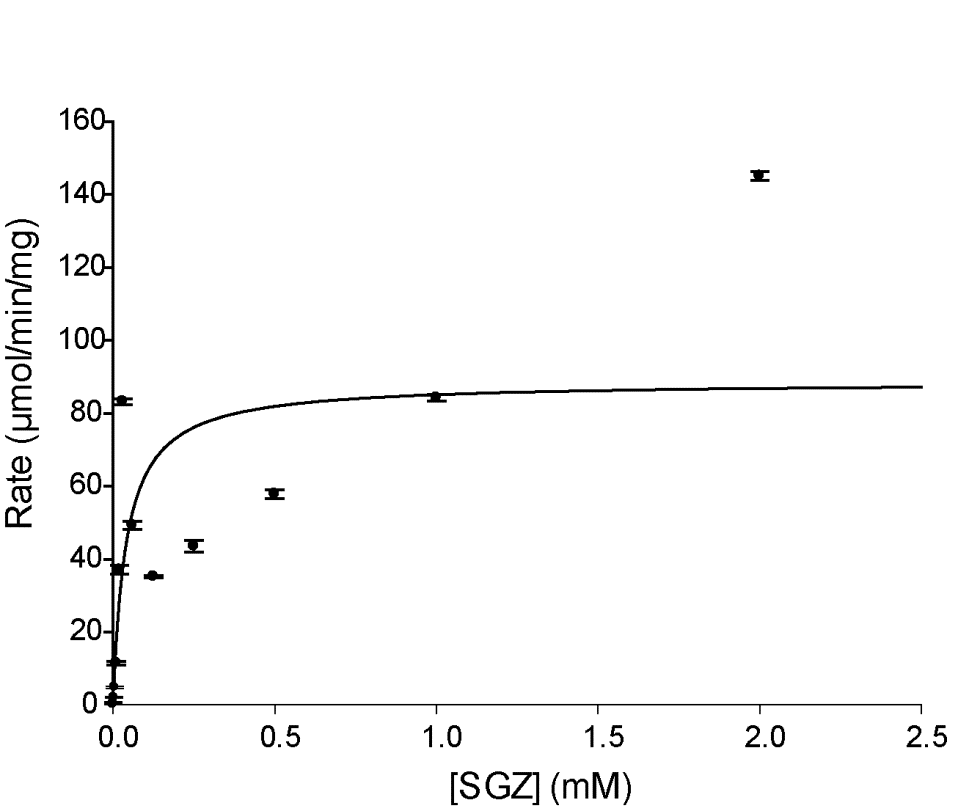


C

**Supplementary Figure S4**. Specific activity of *B. coagulans* LMCO at 30 °C in dependency of substrate concentration. (A) ABTS, pH 4.0, 1.7 µg mL^-1^ protein. (B) 2,6-DMP, pH 7.5, 3.9 µg mL^-1^ protein. (C) SGZ, pH 7.5, 3.9 µg mL^‑1^ protein. Dots and error bars: average values and standard deviations of 3 replicate experiments. Solid line: Michaelis-Menten model with substrate inhibition fitted to experimental data with the least squares method in SIGMA-PLOT. Kinetic parameters in graphs were derived from fitted models.


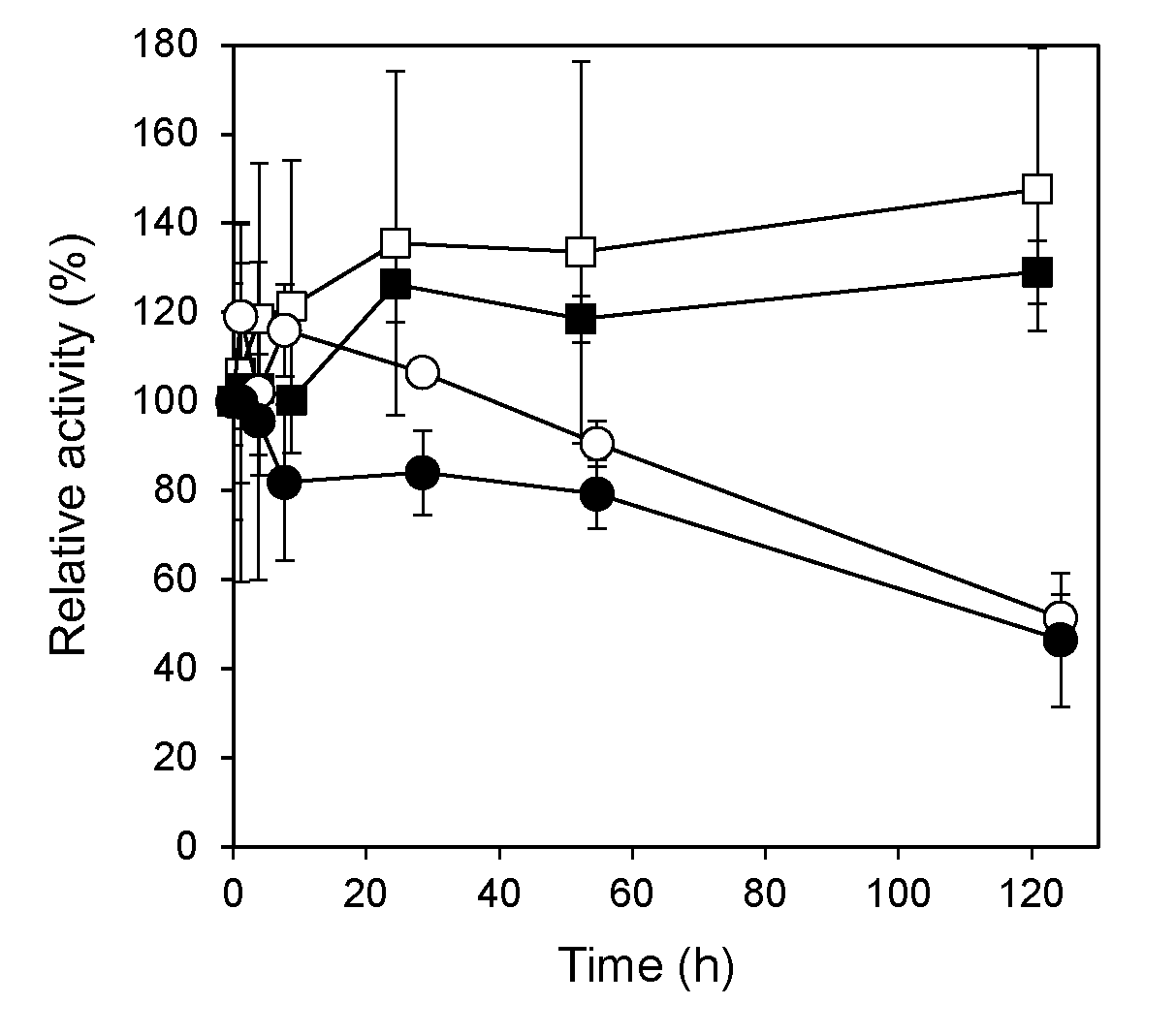

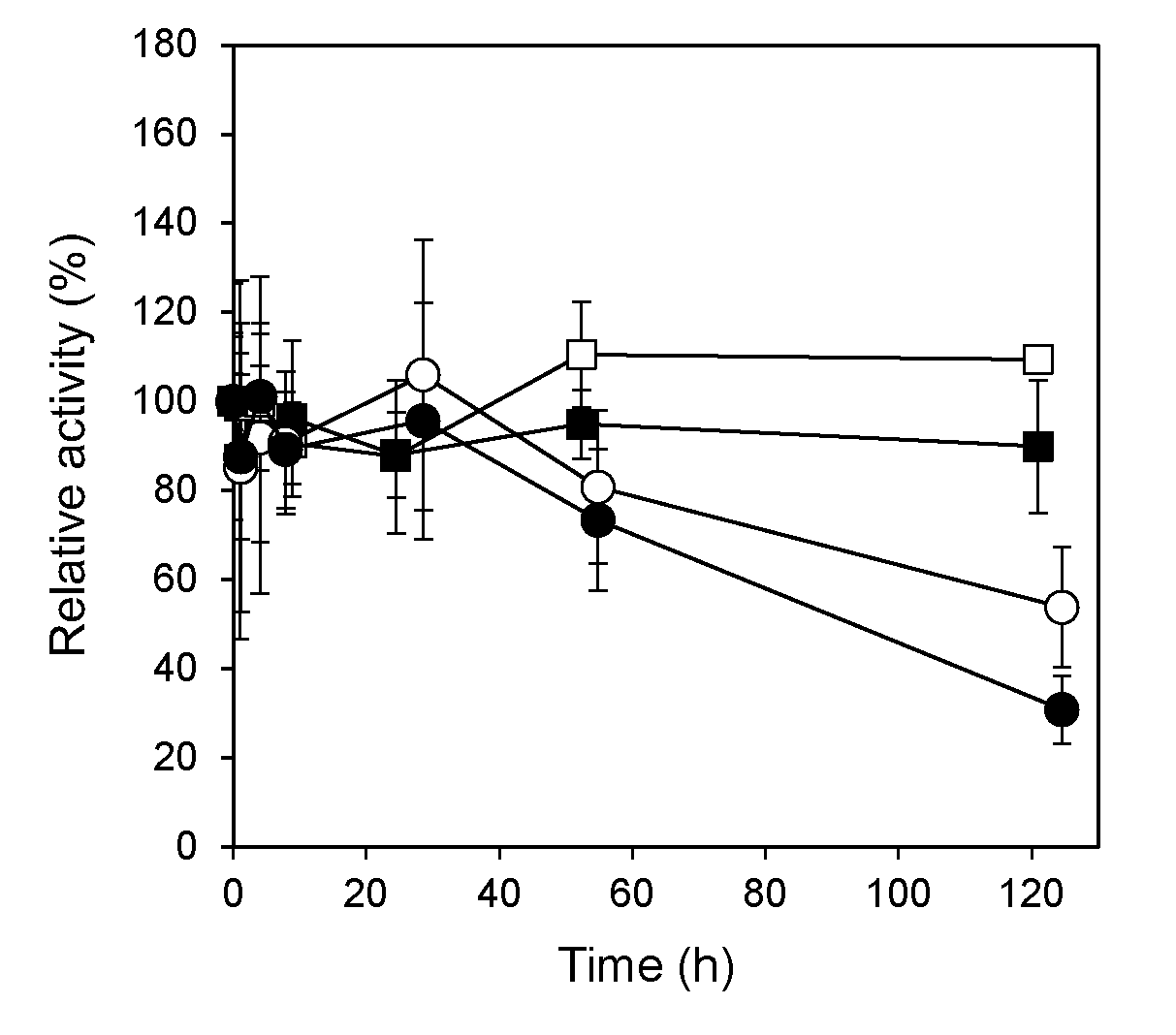


A

B

**Supplementary Figure S5**. Stability of *B. coagulans* LMCO at alkaline pH with and without co-solvent. (A) pH10 (B) pH 11. Similar amounts of heat-purified enzyme were added to Teorell-Stenhagen buffer of the respective pH and samples were withdrawn at different time points and analyzed for relative activity (assay conditions: tartaric acid buffer pH 4, 0.5 mM ABTS, room temperature, rate of absorbance change measured at 420 nm). Squares: incubation at room temperature, circles: incubation at 45 °C; open symbols: buffer without solvents, closed symbols: buffer with 25% v/v DMSO.

Supplementary Methods

**PCR primer sequences and construction of plasmids.** In order to directly compare previously characterised *B. pumilus* CotA (NCBI reference sequence ZP_03054403) to *B. subtilis CotA* expressed from pLOM10^23^ and *B. coagulans* CotA, the full-length gene was subcloned from pBpL6^28^ (pQE-60 vector) into pET-22b(+) by PCR amplification using forward primer 5’-CGTACATATGAACCTAGAAAAATTTGTTGACG-3’ and reverse primer 5’-TGACAAGCTTTTACTGGATGATATCCATCGGC-3’ (restriction site *Nde*I and *Hin*dIII underlined). The 1542-bp PCR product was double digested by *Nde*I and *Hin*dIII and ligated into the respective restriction sites of pET-22b(+), resulting in pBuL.

As example for an alkaliphilic CotA-type enzyme^31^ we included *B. clausii* LMCO in our study (NCBI reference sequence YP_176145.1). The full-length *cotA* gene was PCR-amplified from genomic DNA using forward primer 5‘-CGTCATATGgaattagaaaaatttgtcg -3‘introducing an *Nde*I site with the start codon, underlined) and reverse primer 5‘-CCAGAATTCTCATCGCCTTTCCTTCTCTG-3‘ (introducing an *Eco*RI site after the native stop codon, underlined). The 1545-bp PCR product was double digested with *Nde*I and *Eco*RI and ligated into the respective restriction sites of pET-22b(+), resulting in pBaL.

The putative LMCO gene of *B. coagulans* (NCBI reference sequence YP_004860005.1) was PCR-amplified from genomic DNA using the forward primer 5‘-CGTCATATGAGCCCAAATTTAGAAAAG-3‘(introducing an *Nde*I site with the start codon, underlined) and the reverse primer 5‘-CCAGAATTCttaatccaaaagggggc-3‘ (introducing an *Eco*RI site after the native stop codon, underlined). The 1584-bp PCR product was double digested with *Nde*I and *Eco*RI and ligated into the respective restriction sites of pET-22b(+), resulting in pBoL.

A synthetic gene encoding the putative LMCO of *G. forsetii* in full length (NCBI reference sequence YP_861212.1**),** with codons optimized for *E. coli* K12, was obtained from Eurofins MWG Operon gene synthesis service (Ebersberg, Germany). The gene was cloned in frame without any additional amino acid codons into the *Nde*I and *Eco*RI sites of pET-22b(+). Variants of *G. forsetii* MCO which were truncated N-terminally at predicted signal peptide cleavage sites were constructed by PCR amplification and cloning into pET-22b(+) using either forward primer 5‘-GCATACATATGGGCTTCTATGTCATGCCGTTC-3‘ (removal of amino acids I2-A20, pGoL2) or 5‘-GCATACATATGGATACGAGCACTCGCCATCC-3‘ (removal of amino acids I2-S33, pGoL3) and reverse primer 5’-CGACGGAGCTCGAATTCTCA-3’ (restriction sites *Nde*I and *Eco*RI underlined).

A putative, full-length LMCO gene of *S. pristinaespiralis* (NCBI reference sequence ZP_06908025.1) was PCR amplified from genomic DNA with forward primer 5'-CGTGTCCATGGACAGACGGAGCTTCAACC-3' (introducing an *Nco*I site around the native start codon, underlined) and reverse primer 5'-CCATCGAAGCTTAGTGGTCGTG GCCCTCC-3' (introducing a *Hin*dIII site after the native stop codon, underlined). The 1010- bp PCR product was cloned into the cloning vector pJET/Blunt (Fermentas) by blunt end ligation, resulting in pSpL1. *S. pristinaespiralis* LMCO without the predicted signal sequence was PCR amplified from pSpL1 using forward primer 5'-CGTATCCATGGCACCGGCC CCGGCGAAG-3' (removal of amino acids P2-A41) and the reverse primer described above. The 893-bp PCR product was double-digested with *Nco*I and *Hin*dIII and ligated into the respective restriction sites of the medium copy number, IPTG inducible expression vector pQE-60 (Qiagen, Valencia, USA), resulting in pSpL2. An *Nco*I-*Hin*dIII fragment of pSpL1 encoding the full-length LMCO sequence was sub-cloned into the expression vector pQE-60 using the same restriction sites, resulting in pSpL3.

A putative LMCO gene of *M. tractuosa* (NCBI reference sequence YP_004054188.1) was PCR amplified from genomic DNA without the sequence encoding a predicted signal peptide (i.e. without amino acids E2-C32) using forward primer 5‘-GCATACATATGGACAGTCAATCTTCTACTACAGC-3‘ (introducing an *Nde*I site with the start codon before the D33 codon, underlined) and reverse primer 5’-GGACTGAATTCTTACACTATTTTAAAATTTCGCATCATTCC -3’ (introducing a *Eco*RI site after the native stop codon, underlined). The 1621-bp PCR product was double digested with *Nde*I and *Eco*RI and ligated into the respective restriction sites of pET-22b(+), resulting in pMtraL.

A putative LMCO gene of *S. linguale* (NCBI reference sequence YP_003391614.1) was PCR amplified from plasmid DNA derived from strain DSM74 without the sequence encoding a predicted signal peptide (i.e. without amino acids N2-G26) using forward primer 5‘-GCATACATATGTGCAATACCCACGATATGTCG-3‘ (introducing an *Nde*I site with the start codon before the C27 codon, underlined) and reverse 5’-GGACTGAATTCTTAGTTAACCCGGAAGTTGAG-3’ (introducing a *Eco*RI site after the native stop codon, underlined). The 1435-bp PCR product was double digested with *Nde*I and *Eco*RI and ligated into the respective restriction site of pET-22b(+), resulting in pSiL.
